# Translational research in the genomic era: OPPERA study

**DOI:** 10.1186/1744-8069-10-S1-O1

**Published:** 2014-12-15

**Authors:** Luda Diatchenko

**Affiliations:** 1 Alan Edwards Centre for Research on Pain, McGill University, Montreal, QC H3A 1B1, Canada

## Background

Temporomandibular Disorder (TMD) tends to coexist with other chronic pain conditions in affected individuals and is characterized by a report of pain greater than expected based on the results of a standard physical evaluation. The pathophysiology of this condition is largely unknown, the scientific field lacks biological markers for accurate diagnosis, and conventional therapeutics have limited effectiveness. Growing evidence suggests that chronic pain conditions are associated with both physical and psychological triggers, which initiate pain amplification and psychological distress; thus, susceptibility is dictated by complex interactions between genetic and environmental factors [1].

## Materials and methods

The large human study named OPPERA, Orofacial Pain Prospective Evaluation and Risk Assessment Study, measures both phenotypic and genotypic markers in the TMD patients. The phenotypic markers of greatest interest include measures of pain amplification and psychological measures such as emotional distress, somatic awareness, psychosocial stress and catastrophizing. Genetic markers are also measured in a study by genotyping 2,924 single-nucleotide polymorphisms representing 358 genes known to be involved in systems relevant to pain perception [1].

## Results

The OPPERA findings provided evidence for few single single-nucleotide polymorphisms to be associated with risk of TMD [2]. Furthermore, several single-nucleotide polymorphisms exceeded Bonferroni correction for multiple comparison or false discovery rate thresholds for association with intermediate phenotypes shown to be predictive of TMD onset [3] One of the genes on which we focused our initial research efforts on was the epidermal growth factor receptor (EGFR). EGFR is activated by numerous endogenous ligands that traditionally promote cellular growth, proliferation and tissue regeneration. We first identified that SNPs in the gene loci encoding for EGFR and EREG are associated with the risk of chronic TMD in OPPERA and two other independent human cohorts. Subsequent experiments in animal models reveal the functional involvement of these proteins in the pain pathway, show pharmacological and genetic modulation of pain behavior in rodents and Drosophila, and define the relevant signaling pathway. EGFR–ErbB-4 heterodimer activation by EREG produces pain by regulating the PI3K/AKT/mTOR translational machinery and matrix metalloproteinase-9. As a result of these studies, EREG and EGFR–ErbB-4 can be viewed as novel targets for analgesic development.

## Conclusions

Elucidation of the biological mechanisms by which these markers contribute to the perception of pain in these patients will enable the development of novel effective drugs and methodologies that permit better diagnoses and approaches to personalized medicine.

## Disclosures

No financial relationships to disclose.
